# Transforming in awareness of relationship problems due to excessive private education in Korea

**DOI:** 10.1080/17482631.2019.1586624

**Published:** 2019-04-19

**Authors:** Mee-Ra Kowen, Mikyung Jang, Na-Kyung Lee

**Affiliations:** a Department of Child Welfare, Namseoul University, Chungnam, South Korea; b Department of Child Welfare, Namseoul University, Chunan, Chungnam, South Korea; c Department of Child Welfare, Namseoul University, Chunan, Chungnam, South Korea

**Keywords:** Private education, transform, Korean parents, relationship, counselling

## Abstract

**Purpose**: This study sought to use child and parent counselling to investigate aspects of maladjustment in parent–child and peer relationships of children who had been exposed to excessive private education in early life.

**Method**: The Case study method was used for three children and their mothers, analyzing the process of transformation in awareness of the issue of excessive education, based on the content of the counselling session.

**Result**: The process of change in awareness of problems due to their experiences of excessive private education experiences was divided into five domains and 13 categories. The participants showed that they were aware of the problems in their relationship and consequently made changes to their lives.

**Conclusion**: This study found that counselling helped alleviate the aforementioned problems, which suggests the need for preventive interventions on parent–child relationship in the context of the negative effects of excessive private education.

## Introduction

Excessive private education, a social phenomenon, has a long-standing history in terms of its negative influence on children, their families, and society. According to the National Statistical Office (NSO), 80.7% of the primary school students in Korea receive private education (Statistics Korea, 2015), and 53% of five-year-olds starting early private education are exposed to two main types of private education: academic learning and arts and physical education (Kim, Choi, Choi, & Jang, 2016). Although it is difficult to define how much private education is excessive, according to Park (2002) and Baek, Kim, and Woo (2005), excessive private education comprises extracurricular or private education children receive at home or private institutions, including early private education that centres on cognitive stimuli-based excessive academic learning instead of education, which focuses on the developmental characteristics and children’s aptitudes.

**Table I.** Transforming of awareness of private education experience.

Domain	Category
Being obsessed with private education	1. I have to excel academically to become special
	2. Friends are all rivals.
	3. Compensate for the responsibility of parenting through private education
	4. Transfer of the parent’s dream to become the child’s dream.
Recognizing of parent-child relationship problems	5. Disappearance of emotional dialogue in parent-child relationship.
	6. Become an anxious parent and a depressed child
Recognizing of peer-child relationship problems	7. Confusion with what the problem is.
	8. It’s difficult to do well.
Facing limitations or changes in awareness of excessive private education	9. Being number one only brings endless anxiety
	10. Realizing of the greater importance of relationships than academic performance
Transforming of personal identity through recovery of relationship	11. I can express what’s in my heart freely
	12. Learn what’s in each other’s heart
	13. I have been changing because of and within the new relationship

### Relationship problems

According to a report of KEDI (2002), its effectiveness in bringing about positive results such as improvement in school performance is not consistent with empirical evidence, and rather, it fosters negative results; instead of fostering creativity and originality, it centres on repetitive learning of the same content and thereby increases reliance on private institutions.

Research has shown that excessive private education from childhood negatively influences the child’s psychological, emotional, and behavioural development. In various aspects of early private education, children’s externalizing and internalizing behaviour problems significantly increased, which was statistically correlated with the increase in the amount of early childhood private education (Baek et al., 2005). In addition, excessive private education from an early age has been found to negatively influence the parent–child relationship, as it can cause the child to develop anger and hostility towards the parent, as well as peer relationships (Baek et al., 2005; Kim et al., 2016; Woo, Baek, & Kim, 2005).

According to research, mothers have experienced some conflict in their relationship with their children due to forcing them into excessive private education (Min & Bae, 2014). Nevertheless, they felt it was their responsibility as parents to provide private education (Min & Bae, 2014). In other words the mothers have not considered their children’s feelings about their difficulties with private education, which consequently has led to difficulties in their relationship with their children (Min & Bae, 2014).

Research has shown that as children initially experience the short-term benefits of private education, the parents spend more time and money on it; but in time, the children start to complain about attending the private institutes. Eventually they become more inattentive in class and defiant towards their parents. In a vicious cycle, the parents become anxious and coercive in response, exhibiting a negative aspect of the parent–child relationship.

A study conducted by Woo, Kim, Lee, and Kim (2010) on 1,745 mothers of children aged 0–11 years showed that mothers tended to rate their children’s level of development higher than the actual level. This tendency was more prominent in the area of cognitive development. Consequently, they started private education for their children at an age earlier than what they would have considered appropriate (Woo et al., 2010).

Therefore, parents with a controlling and coercive communication style are more likely to push their children to follow their intentions than seek to understand their children’s feelings and behaviour. Thus, by forcing their children to receive private education, which is not appropriate for their psychological and emotional development, they hinder their relational and social development. The result for the children is various psychological and emotional problems. In a study of adolescents and their parents based on data from the National Survey of Children and Youth in 2008, it was found that parenting methods that determine the characteristics of parent–child relationships influenced the effectiveness of private education. The results of the data analysis of 1,130 households with 15–18-year-olds among 6,923 households under the age of 18 years showed that both neglect and overprotection had negative effects on adolescents’ psychosocial adjustment. Private education was also found to have increased the problem of adolescents’ internalizing behaviour (Ministry of Health, Welfare and Family Affairs, 2008).

According to the analysis of the moderating effect of private education on the relationship between emotional neglect, overprotection and psychosocial adjustment, it was found that excessive private education in a situation of neglect was a risk factor that further increased adolescents’ internalizing problem behaviour. On the other hand, excessive private education in a state of emotional neglect also played a role in acting as a positive/protective factor in mitigating the negative effects on the externalizing problem behaviour.

### Child counselling

Child counselling is a process by which children communicate their emotional, social and behavioural problems with counsellors who have professional knowledge and experience (Jang et al., 2007). However, the characteristics of child counselling are different from adult counselling in that a child is fully dependent on adults, parents have absolute influence on the child, and child counselling necessarily involves the parents. Therefore, it necessitates counselling for parents in order to change the role and attitude of parents who influence the child’s present condition (Kim, 2010).

## Purpose of the study

In such cases, the relationship between the counsellor and the child in the context of child and parent counselling is very personal, intimate, and confidential, which involves expressing the pain and deep emotions of both the child and parents. Therefore, during the counselling sessions, the attitudes, behaviours, and the changing processes of children and parents provide important information for understanding their characteristics to not only the counsellor but also the children and parents. In particular, the main goals of counselling are transformation and recovery; it provides an opportunity to understand the child’s emotional, social, and behavioural problems related to excessive private education and the process of change in the parent–child relationship.

Excessive private education is a prevalent social phenomenon in Korea, and its negative effects are well known. This study aims to conduct a qualitative case study on the process of changing perceptions of the effect of excessive private education on the parent–child relationship and the child’s peer relationships that occur during counselling. We also seek to explore the possibility of making a general application of our findings to the excessive private education group.

## Research method

### Research design

This study used the case study method to examine the experience of school-age children and their mothers in order to better understand the effect of excessive private education on both parent–child and peer relationship problems.

Yin (1994) defined a case study as an empirical study that focused on a phenomenon in the real-life context where the boundary between the phenomenon and context was not clear. Stake (1995) described it epistemologically as research that harmonized with the experience of the reader and provided a basis for valid generalizations. Stake also classified case study research into three categories: the intrinsic, the instrumental, and the collective.

In an intrinsic study the case for its own sake is studied, that is, the researcher explores the features of the case. In an instrumental study, the researcher selects a small group of participants in order to examine a certain pattern of behaviour. In a collective case study, data from several different sources are coordinated and focus is more on the generalization of findings to a bigger population (Stake, 1995). Excessive private education and the resulting relationship problems are prevalent in Korea. To identify perception changes in counselling with regard to excessive private education and negative relationship problem and to explore the possibility of generalizing the findings—i.e., relationship improvement due to changed perception—to the excessive private education group, Stake’s case study method is appropriate. At the same time, the study sought to maintain Stake’s intrinsic and instrumental study methods in order to understand the features of each case.

Furthermore, because this method involves the collection of data over a long-term period, researchers should be immersed in the phenomenon or situation related to the case study. Therefore, a high level of understanding may be achieved as a result of the research (Shin, Cho, & Yang, 2004). For this reason, analyses of the cases of children and parents who have received counselling seem appropriate for the characteristics of Stake’s case study method.

### Data collection

Each case in the case analysis consisted of 32 sessions held over a counselling period of nine months. Children were interviewed once a week for 45 to 50 minutes at an appointed time and place. After each session with the child, the counselling session with the parent was held for 15 minutes. In order to fully use the materials from each case, what the parents and children verbally mentioned during the counselling sessions was analyzed. In addition, the analysis unit of the case study approach can be one case or a small number of cases, but the number of variables of interest in each case is generally large, and all variables should be reviewed (Stake, 1995). Thus, all works such as fantasy play, sand pictures, and paintings made during the counselling sessions were used as supplementary materials for parents and children’s comments. Through counselling, the researcher, child, and mother opened themselves to the problematic situation or event in the past related to private education and developed an understanding of its meaning.

### Rigour of research

To ensure feasibility and credibility, the researchers strove for persistent observation and prolonged engagement during the entire counselling process (Lincoln & Guba, 1985). Such efforts were consistent with the basic principles of counselling: the optimal way to reach a conclusion is through the participant’s statements and involvement, while excluding the counsellor’s subjective involvement as much as possible (Kim, 2016). The study also relied on Lincoln and Guba’s naturalistic interpretation, and thus used the criteria of credibility, transferability, dependability, and confirmability (1985).

For credibility, the study drew on information from multiple sources, including video records of counselling sessions, counsellors’ subjective statements, child participants’ drawings, sandpictures, and triangulation with peer supervisors. In the last session, the researchers discussed the sandpictures and the issues that came up in the counselling sessions with the participants. As for transferability, the researchers employed the technique of thick description so that not only they, but also the participants and readers, could relate to the findings.

And for dependability and confirmability, the study was subject to peer supervision involving mental health professionals, who checked the content and process of counselling, and also a discussion involving two external researchers, who examined data categorization and key themes. In addition, debriefing sessions (Lincoln & Guba, 1985) were held to refute the idea that the problems faced by the participants stemmed from excessive private education, an attempt aimed at eliminating the researchers’ own prejudices and preconceptions.

### Interpretation

The study relied on categorical aggregation, direct interpretation, and naturalistic generalization (Creswell, 2007; Stake, 1995) for data interpretation. In other words, the study attempted to derive meaning by making direct interpretations for individual cases using categorical aggregation and direct interpretation. The research collected instances and found meaning from repeated phenomena until it could be seen that various cases belonged to a single type.

The researchers also employed thick description to draw naturalistic generalizations, through which the narratives connected to the relationship between excessive private education and relationship problems were portrayed as things of our sensory experiences. Doing so allowed the reader to reach conclusions through personal engagement or by vicarious experience. This approach gave vicarious experiences to the reader by adding their experience to cases, and even modified existing generalizations about such cases (Stake, 1995). The process produced five domains and 13 categories for the study.

### Ethical consideration

This study was approved by Namseoul University’s Institutional Ethics and Life Committee (Code: NSU-161,223–01). Before conducting the counselling, the participants of the research were provided with the research description in person regarding the research purpose and method, the principle of anonymity and confidentiality, and participants’ rights and freedom to withdraw from the study, and their verbal and written consent were obtained. They were explained that even after agreeing to participate in the research, they could withdraw at any time during the research process. It was assured that all transcriptions of the counselling sessions would be safely incinerated at the end of the research and the audio-recordings of the sessions would be stored safely by the researcher personally, and that the contents of the case may be shared with the collaborating researchers for analysis. Finally, the ethics of anonymity and confidentiality were strictly maintained by protecting the names of the region, counselling centre, and child in order to keep the individual’s personal information from being exposed in the content of the transcribed case study.

## Participants

Excessive private education in Korea is most pervasive in the capital city of Seoul and some parts of Gyeonggi province. From these two areas, the researchers chose three districts especially known for a zeal for education: Gangnam and Seocho in Seoul, and Bundang in Gyeonggi province. The study then picked three child-family counselling centres from these districts, and ultimately selected three parent–child pairs who requested counselling to resolve their parent–child relationship issues and the child’s peer relationship issues. Among 10 parent–child pairs who well represented the excessive private education group, three pairs that agreed to provide information were selected.

The selected children and mothers meet the following conditions: the child is a primary school student between 7 and 12 years of age; private education began in preschool; the emotional, social, and internalizing or externalizing problem behaviour were expressed due to the stress of private education; and the participants of this study had provided written consent to participate.

All three children had taken more than three private education classes in academic learning and arts and physical education until the beginning of the counselling session; in particular, it was found that they received private education for language and cognitive domains as infants.

The following descriptions of the study participants were based on the mothers’ reports during the counselling sessions, including intake session.

### Child A and mother

The child in Case A was a 10-year-old girl in fourth grade of primary school. When she entered the fourth grade, she was afraid to go to school complaining that her classmates were badmouthing her mother and bullying her. For this reason, she consulted a psychiatrist, who recommended counselling for peer and parental relationship problems.

According to the child’s mother, the client (child) was smart and compliant in the beginning of the first grade, and the mother was the representative of the school’s mothers’ association; thus, there were no problems in the child’s school life. In the second and third grades, although the child did not get along with her peers, she did not face any major conflicts. The mother thought that her child was gifted when she observed her child memorizing an entire English sentence that she had heard only once. Furthermore, the child’s homeroom teacher always told the mother that the child learned English perfectly and she would succeed in anything she pursued and that she was a nice and well-behaved child. Seeing her child receive much positive attention from her homeroom teacher, the mother began to devote herself to providing more private education for her child.

The child’s classmates disliked working with her during the assorted activities class, which required participation from all students. For this reason, the child had considerable difficulty in academic performance. At the start of the counselling session, she was seen talking to herself while playing when she came home. The more she found it difficult to get along with her peers at school, the more she displayed an inclination to obsess over friends. She told her mother that she was always a loner and her classmates hated her and that she wished she had at least one “best friend” or “best pal.”

Her parents were of the same age, and she had a six-year-old sister. The father reported that he had a gentle and amicable temperament and treated the children warmly, while the mother being short-tempered was unable to accommodate the children well. Due to the mother’s career, the child was raised by her maternal grandmother until she was six years old, although the mother brought the child home after work. During this period, the mother did not have much time to play or communicate with her children because of her strong desire to achieve at work.

### Child B and mother

Case B was a 12-year-old girl in sixth grade of primary school. The mother sought counselling with the chief complaint that the child was distracted. Specifically, the main complaint was that “She gets called on a lot because she cannot sit still in class, and instead of paying attention to the teacher, she does something else.” According to her mother’s report, “punishment has become more frequent because she would grumble when scolded for lying or throwing tantrums about finishing her homework.”

The child had been humiliated and excluded by her peers because she did not do well in English despite attending an English institute. She spent a lot of time alone because she could not have a good relationship with her peers at school. At that time, instead of encouraging or comforting the child, the mother condemned and punished her severely and forced her to learn English even harder. Until the beginning of the counselling, the child spent much time in private education for gifted learning. She studied English, mathematics, swimming, singing, music, essay writing, and used three home study workbooks; her private education had been begun at age one.

Currently, the child was residing with her mother, her aunt, and a friend of the aunt. The unemployed and single aunt mainly raised the child. The father of the child was financially negligent, with a large gambling debt, and was laid off from his job. However, even after he lost his job, he did not get a new job, and their marital conflict intensified. They had been separated for seven years.

The mother of the child had a strong sense of responsibility and was very devoted to her child; although her workplace was far from their residence in Seoul, she commuted long distance because of her child’s education. According to the mother’s report, her personality is meticulous, organized, and calculating but she is also very critical and controlling of her child and is inclined to get angered easily, being hot-tempered and impetuous. Although she had a strong desire to raise her child well, in reality, she was strict, controlling, and achievement-oriented in terms of her direct relationship with the child.

According to the psychological evaluation at the start of the counselling session, while the child had a strong desire for intimate relationships and affection, the mother assumed a very strict and critical attitude towards her child’s underachievement and academic failure because she tried to compensate for the burden of having to raise the child as a single mother and the guilt regarding the confusing family environment through academic achievement via private education. More specifically, she was indignant and condemning of her child’s failure to live up to her expectation especially since she had offered her child an opportunity for education that she could not have in the past.

### Child C and mother

The child in Case C was an 11-year-old girl in fifth grade of primary school. The maternal grandmother sought counselling when the child developed motor tic disorder (blinking her eyes, flaring nostrils with open mouth) a year and a half ago. The mother reported that the child had often been praised for her outstanding performance in academics, English, sports, and music, especially compared to her peers; however, when she did not receive praise, she often became angry and weepy. The child had a compulsive need to be the first position; the mother expressed that this type of praise was detrimental to the child. The child reported that her biggest concern was performing well on all exams and achieving victory over a certain friend and that she had the confidence to do so. The child lived with her parents, maternal grandmother, and her third-grader younger sister. The maternal grandmother served as the primary caretaker. The grandmother was devoted to parenting, and the parents were in the position of just observing the child’s situation; the mother seemed to be more comfortable with this arrangement than did the father, who felt that he had been deprived of his children due to the maternal grandmother taking care of the children.

The educational zeal of the grandmother was very strong with a tendency of perfectionism, so she carefully supervised the schedule of the child and her sister, and reprimanded the children when they did not do their homework on time. The father worked as an employee trainer at a company; he was neat and family-oriented and took care of everything that the mother could not. Due to his strong attachment to his children, he wanted to know everything related to them and became irate when he was not informed of matters regarding his children. The mother, who worked in consulting, used to leave for work at 7 AM and returned at 10 PM, and most of her thoughts were focused on work. The mother found it more comfortable to watch the children than to intervene in their lives as she tended to focus more on work than on children. The younger sister was considerably stressed due to frequent comparison to the child who was outstanding in all respects; the younger sister often hit her, slapped her in the face, and sometimes fought and pulled her hair. Due to the child’s strong sense of competitiveness regarding academic performance, she regarded her peers as rivals and did not have a good relationship with them. In the preliminary psychological assessment at the start of the counselling, she exhibited immature situational social judgment and social understanding in comparison to her intelligence, and it was reported that she was normally low-spirited with frequent abdominal pain. Thus, it was possible that the child was suppressing her emotions and that her emotional insecurity was manifested through somatic symptoms. Unlike the reports from her mother and grandmother that she was zealous about learning as much as possible despite her demanding and difficult schedule, rarely reporting any difficulty in her everyday life, it was found that she told her father, “I find it difficult that I have to be better than others when I learn this.” The theme that emerged in SCT was her longing for her mother’s affection, and academic achievement was perceived to be the only way to receive her mother’s affection.

## Results

Through counselling, the mother and child were given the opportunity to express their repressed feelings about private education and experienced a moment of mutual communication regarding the previously unrecognized meaning of private education experience. Furthermore, the mother tried to develop an improved relationship through the parent counselling. The story of the mother and child involved a journey of change, sharing of experiences, recognizing the nature of the experience, and taking a better step forward.

Based on the categorical aggregation or direct interpretation method introduced by Stake (1995), the process of change in the awareness of problems due to excessive private education experiences both in child and mother were divided into five domains and 13 categories, as shown in Table1.

### Being obsessed with private education

#### I have to excel academically to become special

The mothers and children of cases A and C held a common belief that they had outstanding inherent abilities and that they would become more special and be recognized more by others through academic achievement. Achieving the desired outcome and receiving positive feedback from those around them reinforced this idea, causing the children to perceive themselves as being special, which in turn led to focusing more on private education.

*You cannot quit if you start. My mother tells me to not go to the English competition or study English if I do not want to, but I do not think I can quit because I have been trying so hard since I was little* (Child A).

*She won a consolation prize at a national English competition without much preparation. People say that it is incredible* (Mother A).

*The people I interact with at work often care about their children’s education. They say that their children’s private education schedule is packed with English, math, gifted learning centre, another foreign language, ballet, music; unless they do these, they can be bullied by friends. Fortunately, my husband and are able to send our children to these institutes, and they are compliant with this arrangement….I have been somewhat bragging at work about my child being number one. I told my child not to study hard, but I think it felt good whenever she came home with that title* (Mother C).

#### Friends are all rivals

The children were characterized by their tendency to view their peers as rivals and evaluated them sensitively in the private education environment. The attitude of considering the relationship between peers in terms of rivalry led them to perceive others as an object of evaluation and set a pattern of sensitive and passive relationships. This has led to a lack of empathetic and emotional interactions in peer relationships.

*I have never lost because I have learned English since I was four years old. I lost in a game of chess with my cousin a while ago, and I wanted to sink through the floor* (Child A).

*I am afraid that my friends will make fun of me tomorrow when I make mistakes in skipping rope and marathon* (Child A).

*Teacher, my friends said that their legs became pretty because of ballet. So, I said that I wanted to practice ballet again and now I am taking ballet. My friend says that she is entering a ballet competition, and she is good in studies and ballet. I think I should do better because my friends are all good* (Child C).

#### Compensate for the guilt and responsibility of parenting through private education

Parents became obsessed with private education due to the belief in compensation for the guilt and anxiety of not being able to care for their children properly due to conflicts between parents, parents’ feeling of deprivation and the desire for success at work.

*I feel that it is my fault that my child has a low self-esteem and her voice is fainter with the lack of confidence. Why is she like that? She needs to be more confident…. and I think I focused more on her academics then* (Mother A).

*It is not that I do not understand the difficulty that my child has to go through because of more studies and homework than when I was younger. But my child is not the only one studying a lot. Even if she studies like this, she might not make it to college or get a job and will have a hard time making a living. I need to raise my child all alone by myself—what can I do?* (Mother B).

#### Transfer of parent’s dream to the child

There was a tendency for the parent’s unresolved issues, guilt, and frustrated desire for achievement while growing up to become generational. For this reason, instead of focusing on the emotional relationship of the family, the parent focused more on the external environment such as private education.

I think my desire for her to be a child praised by people stems from my own desire to be praised (Case A mother).

I would not have met someone like my husband if I had gone to a private institute and studied more at school while I was in school. What I am most worried about is that she will come to resemble her father who is lazy, irresponsible and a liar. I’ve failed in marriage and the burden of raising a child without a father is too great. And I don’t want my child to live like me. So I live here even though it takes me 2 hours to get work (Case B Mother).

When I was young, I was mostly in the second place. I’ve never been able to be in the first place. My mother was so upset that I could not be upset. But in fact, I was extremely upset and my pride had been injured. I even wanted to go to a school in the countryside to be the first in class. I was hoping for my child to be in the first place because I could not be in the first place (Case C mother).

My grandmother wanted my mom to become a professor since she was a child. So even though the grandparents were somewhat poor, the grandma determined and sent her children to a private school. But she did not become a professor. So my mom wants me to be a professor or more than that (Case C child).

### Recognizing of parent–child relationship problems due to excessive private education

#### Disappearance of emotional conversation in parent–child relationship

The mother started to recognize that her goal-oriented thinking began in order to maintain the academic capabilities she believed that her child was able to achieve through private education so far. She realized that due to this, it was hard to transition from the mindset that quitting private education seemed to diminish the achieved goal; emotional communication between the parent and child had ceased, and commanding and incidental dialogue had become commonplace. The child also began to recognize that her emotional dissatisfaction with her parents manifested in various forms.

*I always seemed to have had some kind of agenda in telling the children what to do and was always explaining it* (Mother A).

*I used to often get angry before. I think I was often very angry with my child. But what is really scary is that even when I was angry, my child did not respond* (Mother B).

*Reading comic books is the only way for me to relieve stress. When I come home, it really annoys me that I have to do my homework when I really want to read a comic book. It’s 9 o’clock in the evening when I come home after attending private institute after school. When I try to read a comic book even for 10 minutes, my mom comes running and asks me to do my homework. I feel as if I have a black cloud hanging over my head when I have to do homework after studying all day* (Child C).

#### Becoming an anxious parent and a depressed child

Mother’s anxiety and deficiency caused her to focus on the child’s academic achievement, and this led to excessive private education, which led to considerable mental suffering for the child. Due to the mother’s obsession with private education, even more coercive control methods were used to control the child for a long time. Learning under compulsion resulted in reduced motivation to learn, leading to depression and a sense of being incapacitated; the mother also began to feel incapacitated.

*When I saw my child develop tics, I said, “You have to fix it. You really look stupid. You look like a total idiot.” She really looked like an idiot. If this child did not do well academically, I would have had a very hard time. Her tics seemed to worsen whenever she watched TV or read a comic book but stopped when she studied* (Mother C).

### Recognizing of peer–child relationship problems due to excessive private education

#### Confusion about what the problem is

The mothers began to think about the actual problem in peer relationships. The children had been bullied by their peers; thus, they felt ignored, depressed, and alienated, and the children began to express their feelings more intensively. The mothers began to realize that their obsession with private education since infancy had brought about self-centred mindset and hindered the child’s social development due to low-empathetic abilities, which in turn led to immaturity and frustration in peer relationships.

*I do not think that I have raised my child very strongly; therefore, if I could go back to her childhood, I would like to raise her again. I got angry just recently because she was diagnosed with scoliosis at the hospital, and I think she got this from hunching over because she was being bullied by her peer group* (Mother A).

*I cannot trust my friends. They seem to talk about me behind my back. They ask me to be their best friend but it seems that they are doing it to keep me from other friends* (Child A).

*People around her have told her “you are a genius,” “you are a gifted child, and she really thought that she was a genius; she did not play with other kids at kindergarten saying that they are insignificant. And even now, she looks down on other kids and still does not get along with them* (Mother C).

#### It is difficult to do well

The children found their long-term coercive private education and the competitive environment difficult; thus, they considered it difficult to be better or more academically accomplished than others.

*My friends do not even like getting a score higher than 80. It is not 100. I am worried that I would not be able to go to college without studying like this. My mom went to college and has a good job but she does not look happy. Mom and aunt sigh almost every day* (Child B).

*My level is different from (that of) my neighbourhood academy. They still teach fifth-grade level English and math, but I am learning middle school math. There is a school bus, but commuting 30 minutes back and forth every day was too hard so my grandmother takes me to the private institute. I am the first in my neighbourhood, but when I go there, I get very nervous because everyone does well*. (Child C).

### Facing limitations of excessive private education and changes in the awareness of excessive private education

#### Being number one only brings endless anxiety

The children were able to acknowledge their insecurities and low satisfaction in the counselling process; they realized that they were seeing others as subjects of evaluation and began acknowledging themselves in the process of striving for a goal rather than a lofty goal of being first.

*I am afraid that if I quit studying even a little, I will mess everything up so I cannot stop…… stop…….I won a prize in the piano and inline-skating competitions. But after winning, I do not want to do them anymore* (Child A).

*I got a score of 97.5 on the math test this time. The lower score was 97.4. I was very nervous and anxious. I felt like crying when I did not get the first place. Next time, I might not be able to come first. But that is okay* (Child C).

#### Realizing of the greater importance of relationships than academic performance

The child and mother began to recognize the limitations of private education and began to improve their relationship. They discovered an urge to interact emotionally with others while recognizing the lack of empathic interactions within the family, and efforts were made to improve the lack of sympathy. In particular, the need for emotional ties was linked to emotional sensitivity and resulted in satisfaction with the relationship.

*These days I feel very sorry every time I see my child, for not spending enough time with her and getting to know what is in her heart, for being too ambitious with my child at the compliments of other people* (Mother A).

*I came back from the Chinese language camp yesterday. I shared a room with a friend who did not clean and was noisy. I wanted to ask for a room change but after talking to her at night, I found out that she had the same worries as I had regarding the relationship with friends; we then became close friends while talking about the same worries by the time we returned* (Child C).

### Transforming of personal identity through recovery of relationship

#### I can express what is in my heart freely

As the mother came to focus on the relationship with her child, the child was able to experience a positive relationship with the mother through the acceptance and attention of her mother. This enabled the mother and child to experience emotional satisfaction, and as the child perceived the mother as a safe subject/target, she was able to express her desires freely. Furthermore, in her peer relationship, as she began to develop an emotional, intimate relationship from a superficial relationship, which was previously only considered competitive, the child changed into a more active and self-confident self.

*My child has changed a bit. She chatters about little things that happened with her friends like a passing story. Previously, whenever I asked about her friends when I was worried, she always said “nothing much” or “everything is alright”* (Mother A).

*I was invited to a game by a friend who is good at games. It was awesome. I am flattered…. I share secrets with my friends these days* (Child A).

*My mom told me about dad for the first time. I was always anxious and curious, but I could not ask. To be honest with you, I did not want mom to get a divorce. But I told her that it was mom’s life and since that was what she wanted, she should do it and that I was okay. And I found out why she was obsessed with my studies and homework* (Child B).

*I told my mom that I wanted to eat with my friends again. More specifically, I told her that I wanted to have a birthday party at home, and she took some time off from work to throw me a birthday party* (Child C).

#### Learn what is in each other’s heart

As the mother and child recognized the importance of emotional intimacy in a relationship, they felt the needs and emotional intimacy of the relationship genuinely. Furthermore, as the mother withdrew her sense of inferiority due to her child, their relationship developed into one in which the child could communicate about everything comfortably with her mother and in turn gave the mother the opportunity to re-establish her role as a parent.

*I have always had a one-way conversation with my children of ordering and disciplining them. From now on, I would better attend to her when she talks to me. I think she is very immature in communicating with others because I have not been listening to her* (Mother A).

*When I was little, my wants had never been fulfilled properly, and after marriage, I had hoped to have everything I expected from my husband to be fulfilled through my child. Whenever she called, instead of communicating with her emotionally, I only checked her homework. My sister, her friend, and I are all diligent and have a strong sense of responsibility, and no matter how hard we work on something without any break, none of us have ever laughed or felt happy. As much as I did not want to live with my own parents, I do not want my child to live like us. When we adults go home, we want to do nothing, space out, and just watch TV; imagine how hard it must have been for my child to do all that homework* (Mother B).

#### I have been changing because of and within the new relationship

The child recognized the self-discordance she experienced when focusing on private education; she realized her true need, and through it, expressed positive changes through a story. The child came to realize her true self through experiences other than private education, and change in perception, which enabled the mother to re-establish her role as a parent, had been achieved.

*An egg becomes a caterpillar, a caterpillar a pupa, and like a caterpillar, I think it is hard for me. I am having a hard time like the caterpillar, but I hope I can transform beautifully into a new me like the caterpillar. And since I am a human being, after the transformation, I will live much longer than a caterpillar. Then I will become a good mother later* (Child A).

*I think people should get parenting education before having a baby. I was a parent on an impulse without knowing the proper method of parenting. I would raise her well if I could raise her again. For her first-year birthday party, I made a big banner with her face printed on it. I only focused on unimportant matters like that but did not consider what was in her heart. I really regret raising her so inflexibly, and for being so rigid and coercive with her* (Mother A).

*I decided to get divorced. I was afraid of others finding out that I have failed in marriage, but I am going to have to straighten things out. I want to be somewhat free from the eyes of others. When I told my child, she cried a lot. Then after not talking to me for two days, she hugged me tight and told me to do what I wanted to do yesterday. When my child hugged me, I was in tears, and it was really hard for me. I thought she had grown up so much; I was so grateful that she had grown up so well* (Mother B).

*I have made a lot of friends. I am going to a friend’s birthday party this weekend. My friends say that I am trustworthy. I know a lot of things and am a lot of fun because I am a wizard on celebrities. And I am good at making things. When I make things, my friends want to buy them. My mom tells me not to sell them to my friends but I enjoy it. My friends like it too* (Child B).

*English has become more interesting now that I started studying English with my mom. I do not have to take a test anymore so I feel so much more comfortable. I am going to ask my dad to teach me math and not attend the math institute. It is more comfortable and better to stay at home* (Child C).

*I was supposed to be home around 9 p.m. previously, but I changed my mind to participate more in parenting; it is considerable pressure but now I want to see my children more. This must be what it feels like to be a mom. What does it feel like…it is endearing…it is deeply moving…I wonder why I spent so much time like this when I remember how I used to spend time with my children when they were around three years old. We have more time to spend with family now that we decreased the number of private education classes the children attend; although we fight, getting to know the children’s minds is fun* (Mother C).

## Discussion

This study sought to use child and parent counselling to investigate aspects of maladjustment in parent–child and peer relationships in children who had been exposed to excessive private education in early life. Furthermore, an in-depth analysis of the process of changes in awareness regarding the issue of excessive education was performed based on the content of the counselling sessions. According to the reports of the parents who participated in this study, their children had received various types of private education since infancy. Since then, the children had experienced parental and peer relationship problems due to the parents’ obsession with coercive forms of private education. Furthermore, they expressed various emotional and behavioural problems; these findings are consistent with the results of previous research on relationship problems due to excessive private education and its pathological aspects (Baek et al., 2005; Min & Bae, 2014).

Five domains and 13 categories were derived in this study. The first domain was the cause of obsession with excessive private education. There were three categories in this domain: to be special, to win in competition with others as they were considered rivals, and to compensate for parents’ anxiety and guilt that they were not bringing up their children properly. The categories in the first domain are the ones that most Korean parents with excessive educational zeal often tend to experience while obsessing over private education. By depending on academic achievement through private education, parents feel that their children become special, and that they have fulfilled their parental responsibilities. Mothers’ anxiety and guilt were associated with marital problems and deprivation stemming from their experiences growing up.

The second and third domains are related to the recognition of problems in parent–child and peer relationships. During the counselling sessions, as the counsellor built a rapport with the child, they began to divulge negative emotions regarding private education. Parents and children began to realize that excessive private tutoring led to problems in parent–child relationships as well as peer relationships.

They began to realize that parents’ pressure and coercive parenting to continue excessive private education and maintain high levels of academic achievement reduced the emotional interaction between parents and children and resulted in anxiety in parents and feelings of depression and helplessness in children. This led to a vicious cycle of communication breakdown. These results are consistent with those of previous studies that coercive parenting attitude regarding private education leads to communication breakdown in the parent–child relationship (Min & Bae, 2014) and depression and feelings of helplessness in children (Woo et al., 2010). Furthermore, the participants of the study came to realize that coercive private education from early childhood resulted in an overly competitive attitude, thus depriving children of diverse experiences of peer relationships and impairing the development of mutual communication and interpersonal skills.

The fourth domain deals with the confrontation with the limitation of excessive private education and changes in perception. As the counselling sessions reached the final phase, the parents came to realize that academic achievement through private education could not solve all problems; there were changes in the perception of the parents and their children in choosing relationship over achievement. They were able to acknowledge that even when their goal-oriented efforts were achieved, the children could not be happy due to the lack of relationship and the satisfaction that comes with it. The parents also realized that they had considerable anxiety no matter how much they focused on private education.

The final domain was “rebuilding personal identity and relationship through the restoration of relationship.” In this domain, the restoration of the parent–child relationship was achieved experientially. The mother was able to re-establish her role as a parent and find positive maternal nurturing energy, and at the same time, instead of seeing her child as an extension of her own anxiety and deprivation, she perceived her child as she was. The child could identify her true self through a variety of relationships by making friends of her age and going on family trips, rather than attending private institutes. The mother began to sympathize with her child’s wants and emotions after realizing her lack of empathetic ability, and placed more emphasis on developing an emotional, intimate relationship with the child. Such changes in the mother’s perception allowed the child to experience herself in a new relationship; through this experience, she was able to express herself freely in various relationships.

The findings of this study have several implications. There is a need to understand the negative effects of excessive private education on children’s development in the parent–child relationship instead of regarding them as mere transient influences of private education. A negative social context leads to excessive education; however, changes in social context occur over a long period of time. Thus, the greatest implication of this study is that an intervention that promotes positive changes in parent–child relationships protects children from negative social factors.

Children are placed under the pressure of excessive private education by their parents from early life. However, instead of facilitating the children’s learning, excessive private education cause relationship problems for children and parents. A negative parent–child relationship is a vicious cycle, causing children to experience peer relationship problems, excessive pressure and competitiveness regarding academic achievement and psychological problems that follow. This study found that counselling helped alleviate the aforementioned problems, which suggests the need for preventive interventions on parent–child relationship in the context of the negative effects of excessive private education.

## References

[CIT0001] Baek, H. J., Kim, H. S., & Woo, N. H. (2005). A study on the behavioural problems of preschoolers who experienced early supplementary education. *Korea Journal of Child Care and Education*, 43, 23–11.

[CIT0002] Creswell, J. (2007). *Qualitative inquiry and research design 2E: Choosing among five tradition*. Thousand Oaks: Sage.

[CIT0003] Jang, M., Lee, S. H., Cho, E. H., Chung, M. J., Son, K. O., & Yu, M. S. (2007). *Counseling children*. Seoul: Tae-yeong Book.

[CIT0004] KEDI (2002). *An analytical study on the meaning of public education and the publicness of education*. Seoul: Korea Educational Development Institute.

[CIT0005] Kim, E. Y., Choi, H. M., Choi, J. E., & Jang, M. (2016). *The actual condition education for infant and toddlers II: Centered on 2 and 5 year old*. Seoul: Korea Institute of Child Care and Education.

[CIT0006] Kim, J.-I. (2016). *Group counseling for children and adolescents*. Seoul: Yangsoewon.

[CIT0007] Kim, S. H. (2010). *Child counseling*. Seoul: Hakjisa.

[CIT0008] Statistics Korea. 2015. Survey on Private Education Expenses in 2014, 4. Retrieved from http://kostat.go.kr (Accessed on 21 January 2017).

[CIT0009] Lincon, Y. S, & Guba, E. G. (1985). *Naturalistic inquiry*. Beverly Hills, CA: Sage.

[CIT0010] Min, S. O., & Bae, J. H. (2014). Mothers’ perceptions of and experiences about children’s private education. *Journal of Child Education*, 23, 263–285.

[CIT0011] Ministry of Health, Welfare and Family Affairs. (2008). *Census for children and adolescents*. Seoul: Ministry of Health, Welfare and Family Affairs.

[CIT0012] Park, C. O. (2002). Relationships among early childhood extracurricular experiences, learning attitudes and academic self–Concept of elementary school children, Master’s thesis. Kon–Kuk University of Education: Seoul.

[CIT0013] Shin, K. R., Cho, M. Y., & Yang, J. H. (2004). *Qualitative study research*. Seoul: Ewha Women’s University Publishing Department.

[CIT0014] Stake, R. (1995). *The art of case study research*. Thousand Oaks: Sage.

[CIT0015] Woo, N. H., Baek, H. J., & Kim, H. S. (2005). The cognitive, emotional, and social characteristics of preschoolers in early childhood private education as perceived by private kindergarten headmasters. *Early Education Studies*, 25(1), 5–20.

[CIT0016] Woo, N. H., Kim, Y. S., Lee, E. J., & Kim, H. S. (2010). Mothers’ attitudes toward child development and private education, and the actual state of private education in Korea. *Journal of Eco–Early Childhood Education*, 9, 81–104.

[CIT0017] Yin, R. K. (1994). *Case study research: Design and methods* (*4th ed.* ed.). Thousand Oaks: Sage.

